# Out of hospital cardiac arrest: experience of a bystander CPR training program in Karachi, Pakistan

**DOI:** 10.1186/s12873-022-00652-2

**Published:** 2022-06-03

**Authors:** Uzma Rahim Khan, Umerdad Khudadad, Noor Baig, Fareed Ahmed, Ahmed Raheem, Butool Hisam, Nadeem Ullah Khan, Marcus Ong Eng Hock, Junaid Abdul Razzak

**Affiliations:** 1 Present Address: Department of Emergency Medicine, Aga Khan University, Stadium Road, P. O. Box 3500, Karachi, 74800 Pakistan; 2 Harvard T.H. Chan School of Public Health, Boston, USA; 3 Department of Emergency Medicine, Singapore General Hospital and Duke National University of Singapore, Singapore, Singapore; 4 Department of Emergency Medicine, Weill Cornell Medicine, NewYork, USA; 5 Center of Excellence for Trauma and Emergencies, Aga Khan University, Karachi, Pakistan

**Keywords:** Bystander, CPR, Training, Retention, Pakistan, Out of the hospital cardiac arrest

## Abstract

**Background:**

Nearly 90% of out-of-hospital cardiac arrest (OHCA) patients are witnessed, yet only 2.3% received bystander cardiopulmonary resuscitation (CPR) in Pakistan. This study aimed to determine retention of knowledge and skills of Hands-Only CPR among community participants in early recognition of OHCA and initiation of CPR in Karachi, Pakistan.

**Methods:**

Pre and post-tests were conducted among CPR training participants from diverse non-health-related backgrounds from July 2018 to October 2019. Participants were tested for knowledge and skills of CPR before training (pre-test), immediately after training (post-test), and 6 months after training (re-test). All the participants received CPR training through video and scenario-based demonstration using manikins. Post-training CPR skills of the participants were assessed using a pre-defined performance checklist. The facilitator read out numerous case scenarios to the participants, such as drowning, poisoning, and road traffic injuries, etc., and then asked them to perform the critical steps of CPR identified in the scenario on manikins. The primary outcome was the mean difference in the knowledge score and skills of the participants related to the recognition of OHCA and initiation of CPR.

**Results:**

The pre and post-tests were completed by 652 participants, whereas the retention test after 6 months was completed by 322 participants. The mean knowledge score related to the recognition of OHCA, and initiation of CPR improved significantly (*p* < 0.001) from pre-test [47.8/100, Standard Deviation (SD) ±13.4] to post-test (70.2/100, SD ±12.1). Mean CPR knowledge after 6 months (retention) reduced slightly from (70.2/100, ±12.1) to (66.5/100, ±10.8). CPR skill retention for various components (check for scene safety, check for response, check for breathing and correct placement of the heel of hands) deteriorated significantly (*p* < 0.001) from 77.9% in the post-test to 72.8% in re-test. Participants performed slightly better on achieving an adequate rate of chest compressions from 73.1% in post-test to 76.7% in re-test (p 0.27).

**Conclusion:**

Community members with non-health backgrounds can learn and retain CPR skills, allowing them to be effective bystander CPR providers in OHCA situations. We recommend mass population training in Pakistan for CPR to increase survival from OHCA.

**Supplementary Information:**

The online version contains supplementary material available at 10.1186/s12873-022-00652-2.

## Background

Out of Hospital Cardiac Arrest (OHCA) is a major global health concern and one of the leading causes of death worldwide. In developing countries, it accounts for 10% of mortality [1–4] Globally, it is estimated that on average, less than 10% of all patients with OHCA will survive [5].

In the United States, Resuscitation Outcomes Consortium Registry (ROC), survival rates were significantly higher (43.6%) if layperson-initiated CPR occurred. In the Cardiac Arrest Registry to Enhance Survival (CARES) in the US, automated external defibrillator (AED) use and bystander CPR explained as much as 50.4% of survival variation across 132 counties [6, 7]. In developing countries, where there is a lack of knowledge about bystander CPR, survival rates are dismal. Studies from developing countries have shown varying survival from as low as 0% in Mexico to 2% in Islamabad, Pakistan, and 11% in Karachi, Pakistan [8–10].

The Pan-Asian Resuscitation Outcomes Study (PAROS) represents a concerted effort in prehospital emergency care research to document cardiac arrest epidemiology and ascertain possible outcomes of bystander CPR [11]. With regards to bystander CPR, it is important to recognize that bystanders refer to the population at large,who is responding to the cardiac arrest event before the arrival of an organized emergency response team.. Within Pakistan, numerous studies have affirmed the need for bystander CPR training. One study by Mawani et al. found that amongst a cohort of OHCA patients brought to an Emergency Department, most of the patients (92.9%) had witnessed cardiac arrest out of which only a small percentage (2.3%) received bystander CPR [12]. Another study by Khursheed et al. found that Pakistan had a higher burden of dead on arrival (DOA) patients than other similar resource settings (about 1 to 2 per 1000 visits) and one of the causes they cited was lack of bystander CPR and prehospital care [13].

It is important to reach out to a diverse spectrum of the population and provide them with life-saving knowledge and skills to reduce the burden of OHCA mortality. However, the baseline knowledge of a population is equally important in assessing the impact of life-saving interventions including CPR training.

### Aim of the study

The aim of this study was to determine the retention of knowledge and skills of Hands-Only CPR training among community participants in early recognition of OHCA and initiation of CPR in Karachi, Pakistan.

## Methods

### Study design and setting

We conducted a pre and post-test study from July 2018 to October 2019 in Karachi, Pakistan.

### Participants

We recruited 652 participants that included teachers, ambulance drivers, traffic police officers, patrolmen, and military officers. Ambulance drivers were not trained in emergency medical services in our setting. They were predominantly drivers without any kind of formal training in basic life support. These drivers typically follow “scoop and run” approach instead of “stay and play”. The military officers included Pakistan Rangers, a paramilitary force in Karachi that is conventionally deployed in heavily trafficked neighborhoods, making them potential first responders alongside traffic police. We selected this population to train because all these professions are related to public dealing and they can be mobilized easily in emergencies to act as potential first responders. Their nature of work and training make them more appropriate for the practical application and long-term sustainability of this life-saving procedure. Moreover, given the lack of Samaritan law (protecting those legally who willingly assist individuals in medical emergency) in our setting, this set of population will be actively responding to the OHCA events without much fear.

### Study instruments

Knowledge assessment questionnaires and skill checklists were developed with the help of a literature review of CPR training [14–16] along with expert opinion from emergency physicians.

The knowledge assessment questionnaire consisted of nine multiple-choice and five binary response (Yes/No) questions related to CPR knowledge, attitude, and practice such as “what do you think CPR means, what are the signs of sudden heart arrest, what are the critical components of high-quality CPR, have you received CPR training and what concerns may prevent you from performing CPR if needed” (For further details on the questionnaire, see Supplementary Item 1). Demographics of participants were also asked. All the participants underwent this CPR knowledge questionnaire which took them 25–30 minutes to complete. The questions were read out loud to the participants in the Urdu language. In addition, guidance was provided to the participants who were unable to comprehend the questions.

In addition, CPR performance checklists had three distinctive criteria to assess the participant’s performance that included a participant’s ability to initiate CPR, perform high-quality CPR and discontinue CPR when needed (For detailed information on the checklist, see Supplementary Item 2). The facilitator independently assessed the CPR skills of the participants via a Yes/No checklist.

### Training

The group of 25–30 participants were approached for one-time CPR training sessions. The arrangement of participants was made through appointment, either via phone or email to the participant’s primary organization. The training was conducted based on the availability of the participants during the study period. The training was carried out at the participant’s primary organization or the emergency department of the AKU. Each group of participants received a well-designed 2-hour CPR training based on the American Heart Association (AHA) guidelines. The facilitators were BLS certified and had prior CPR training. The facilitators included emergency faculty, master trainers, medical interns, and emergency medicine residents. There were two leading facilitators with the team of co-facilitators. There were two to five faciltitators per session. First, the facilitator shared information related to CPR in the Urdu language. Second, a video-based CPR demonstration was displayed to participants with a live translation into Urdu language simultaneously for each step of CPR demonstrated in the video. Third, we asked all the participants to demonstrate the steps of CPR independently on the manikins. In addition, we provided scenario-based training to the participants to help them reflect on true situations and enhance their understanding. Moreover, the facilitators provided continuous feedback to the participants regarding CPR techniques while they were demonstrating on manikins. Furthermore, we conducted a post-training evaluation to improve the training experience for participants in the succeeding trainings.

### Assessment

The CPR knowledge assessment questionnaires and skill checklist were completed anonymously. Participants were assessed for knowledge and skills of CPR before training (pre-test), immediately after training (post-test), and 6 months after pre-test (re-test). All the facilitators were trained to use the checklist and rate the performance of participants objectively. The facilitator verbally read out different case scenarios to the participants on drowning, poisoning and road traffic crashes, etc., and asked the participants to perform the critical steps of CPR identified in the scenario on manikins. The facilitator rated the CPR skills of the participants on maximum three attempts while observing the specific skill for at-least 2 minutes. Feedback was given to the participants who failed to perform the correct skills in their attempts. The participants to facilitator ratio ranged from 5 to 10:1 for skill assessment. Each participant was evaluated by a single facilitator and used an uniform checklist approach to minimize inter-rater variability. Moreover, these facilitators were not involved in the analysis of the study.

### Outcome measure

The primary outcome was the mean difference in score on knowledge and skills of the participants related to the recognition of OHCA and initiation of CPR.

### Ethical considerations

This study was conducted after obtaining approval from the ethical review committee of the Aga Khan University (AKU). Written informed consent was obtained from all the participants before enrolment in the study. The confidentiality of the participants was maintained throughout the study and anonymity was ensured by using unique identification numbers for each participant.

### Statistical analysis

All data were entered and analysed using the Statistical Package for Social Sciences (SPSS) version 21 [17]. Descriptive statistics were used for demographic variables and presented as frequencies and percentage. The mean knowledge score was computed for pre, post and retest. Parametric tests were used since the data obtained were normally distributed. We also used repeated measures ANOVA to determine the significant mean between pre, post and re-test. The association between knowledge score and demographic characteristics was analyzed using generalised estimating equation regression. A *p*-value of less than 0.05 was considered significant.

## Results

### Sociodemographics of the study participants

The pre and post-tests were completed by 652 participants, whereas the re-test was completed by 322 participants. Table 1 shows the sociodemographic characteristics of the participants. Most of the participants were male (*n* = 553, 84.8%) and had secondary school (grade 9–10) education (*n* = 284, 43.6%). The majority of the participants (*n* = 404, 62%) were in the age group of 25–44 years. In addition, a majority of the participants (*n* = 257, 64.7%) had no training in CPR before pre-testing. The number of participants dropped to half of the original sample size (*n* = 322) at the time of knowledge retention assessment (re-test). The reduction in sample size was primarily due to military postings that change quarterly.

**Table 1:** Sociodemographic characteristics of the study participants from July 2018 to October 2019

Demographics Characteristics	Pre and Post-test ***n*** = 652	Re-test ***n*** = 322	***P***-value
**Gender**			
Male	553 [84.8%]	286 [88.8%]	0.048*
Female	99 [15.2%]	36 [11.2%]	
**Education Status**			
Primary school	55 [8.4%]	32 [9.9%]	0.823
Secondary school (Grade 9–10)	284 [43.6%]	135 [41.9%]	
Intermediate/ A-Level	141 [21.6%]	73 [22.7%]	
Graduation	120 [18.4%]	53 [16.5%]	
Post-graduate	52 [8%]	29 [9%]	
**Age Groups**			
18–24 Years	115 [17.6%]	50 [15.5%]	0.441
25–44 Years	404 [62%]	196 [60.9%]	
> =45 Years	133 [20.4%]	76 [23.6%]	
**Have you received any prior**^a^ **CPR training?**			
Yes	140 [35.3%]	66 [20.5%]	< 0.001*
No	257 [64.7%]	256 [79.5%]	
**Cadres**			
Military officers	381 [58.4%]	165 [51.2%]	0.043*
Ambulance drivers	92 [14.1%]	60 [18.6%]	
School Teachers	83 [12.7%]	35 [10.9%]	
Traffic Police Officers/ Patrolmen	96 [14.7%]	62 [19.3%]	

^a^CPR = Cardiopulmonary resuscitation

*P value < 0.05 is significant

### Knowledge of CPR at pre, post, and re-tests

On the pre-test, Q13, which is related to the understanding that survival is high when CPR is done immediately received the highest number of correct responses (*n* = 600, 93.9%). Other questions on which participants scored high in the pre-test included (Q10) the understanding that CPR is an emergency procedure that is attempted to restart the heart that has stopped beating (*n* = 571, 89.9%) and (Q12) understanding that CPR is generally continued until the person regains consciousness or is declared dead (*n* = 566, 88.9%). Table 2 shows the frequency and percentage of the participants with correct responses for knowledge assessment questions at pre-test, post-test, and re-test. The retention of CPR knowledge related to the rate of chest compression (Q6) reduced from *n* = 596, 92.4% to *n* = 245, 76.1% followed by (Q7) force that must be applied during chest compressions (from *n* = 411, 63% to *n* = 151, 46.9%) and (Q9) switching roles when performing two-rescuer CPR (from *n* = 571, 87.6% to 167, 51.9%). More than 90% of the participants had knowledge retention for the questions that had binary (Yes/No) responses; questions number 10 to 14 (See Supplementary Item 1 for knowledge assessment questions).

**Table 2:** Score of CPR knowledge among participants from July 2018 to October 2019

Knowledge attributes	Frequency of participants with correct answers		
	Pre-test n [%] ***n*** = 652	Post-test n [%] ***n*** = 652	Re-test n [%] ***n*** = 322
1.What do you think “Cardiopulmonary resuscitation (CPR)” means?	500 [78.7%]	605 [93.8%]	296 [91.9%]
2.Which of the following may be a sign of sudden heart arrest?	5 [0.8%]	51 [7.8%]	19 [5.9%]
3.How can the consciousness state of the individual be determined?	80 [12.3%]	116 [17.8%]	76 [23.8%]
4.How can the absence of respiration be determined?	74 [11.3%]	247 [37.9%]	91 [28.2%]
5.CPR is performed on which part of chest?	368 [58.1%]	563 [87.8%]	282 [87.6%]
6.What must be the rate of the chest compressions?	207 [33%]	596 [92.4%]	245 [76.1%]
7.How much force must be applied during chest compressions?	114 [18.2%]	411 [63%]	151 [46.9%]
8.The critical characteristics of high-quality CPR include which of the following?	34 [5.2%]	134 [20.7%]	46 [14.4%]
9.How often should rescuers switch roles when performing two-rescuer CPR?	177 [28%]	571 [87.6%]	167 [51.9%]
10.Cardiopulmonary resuscitation (CPR) is an emergency procedure which is attempted to restart the heart that has stopped beating.	571 [89.9%]	610 [95.3%]	312 [96.9%]
11.CPR has to be attempted always inside of a hospital and not outside.	523 [82%]	599 [93%]	308 [96%]
12.CPR is generally continued until the person regains consciousness or is declared dead.	566 [88.9%]	621 [96.6%]	305 [95%]
13.The survival rate is high when CPR is done immediately.	600 [93.9%]	629 [97.4%]	318 [99.1%]
14.Is CPR effective in saving life of children?	454 [71%]	623 [97.2%]	296 [92.2%]

### Mean CPR knowledge score

**Fig. 1:**
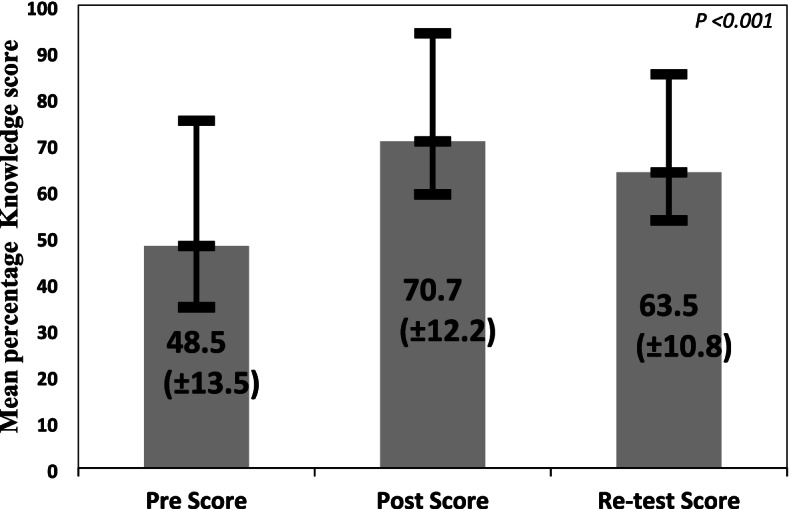
Mean CPR knowledge score (%) at pre, post and re-test (*n* = 322). The mean value of the knowledge score calculated by repeated measures ANOVA has been statistically significant *p*-value < 0.001 between. 1) Pre-test & post-test. 2) post-test & re-test. 3) Pre-test & re-test

Figure 1 shows the mean percentage of the knowledge score at pre, post, and re-test phases. The mean percentage of the knowledge score at pre-test was 48.5 (SD ±13.5) which increased to 70.7 (SD ±12.2) at post-test and decreased to 63.5 (SD ±10.8) at re-test. This was statistically significant (*p* < 0.001). .

### CPR skill performance and retention

**Fig. 2:**
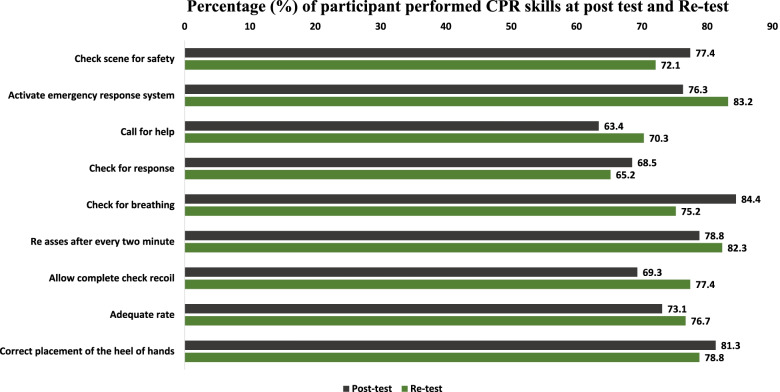
Participants CPR skills performance at post and re-test. The percentage of the participants performed CPR skills correctly at post-test and re-test was computed. The CPR skills assessed are given in y-axis

The CPR skills of the participants were assessed post-training and after 6 months (re-test). Figure 2 shows the percentage of the participants who performed CPR skills correctly at the first attempt at post-test and re-test. Majority of the participants were able to perform skills correctly regarding checking for the scene safety (*n* = 504, 77.4%), checking for response (*n* = 446, 68.5%), calling for help (*n* = 413, 63.4%), and activating emergency response system (*n* = 497, 76.3%). Furthermore, 81.3% (*n* = 530) of the participants were able to place their heels of hands appropriately while performing CPR whereas, 73.1% (*n* = 476) had an adequate rate of chest compressions. The retention of CPR skills slightly deteriorated for placing the heel of hands correctly from 81.3 to 78.8%, checking for scene safety from 77.4 to 72.1% and checking for breathing from 84.4 to 75.2%. Participants performed better on achieving an adequate rate of chest compressions from 73.7% in post-test to 76.7% in re-test and allowing complete chest recoil from 69.3 to 77.4%.

### Generalized estimating equation regression for sociodemographic characteristics

The Generalized Estimating Equation (GEE) analysis revealed that gender and prior CPR training were found to be strongly associated with improved CPR knowledge scores. Table 3 shows the regression coefficients for the GEE model relating CPR knowledge score to sociodemographic characteristics. The females scored on an average of 3.56 points (95% CI: 1.7, 5.4) higher than males. In addition, participants who had prior CPR training scored on an average of 2.61 points (95% CI: 0.8, 4.4) higher than those who had not. The increase in CPR knowledge scores overtime did not vary significantly by age groups and education status of the participants.

**Table 3:** Overall impact of sociodemographic characteristics on the CPR knowledge

Factors	β (95% CI) ^a^
**Intercept**	53.22 (49.7, 56.8)
**Age Group (in years)**	
18–24 (Ref.)	
25 to 44 years	2.78 (0.9, 4.7)
45 Years & above	2.66 (0.3, 5.1)
**Gender**	
Male (Ref.)	
Female	3.56 (1.7, 5.4)
**Education Status**	
Primary School (Ref.)	
Secondary school (Grade 9–10)	2.68 (−0.2, 5.5)
Intermediate/A-Level	4.91 (1.7, 8.1)
Graduation	6.43 (3.2, 9.6)
Post-Graduation	5.03 (1.7, 8.4)
**Prior CPR training**	
No (Ref.)	
Yes	2.61 (0.8, 4.4)

^a^Generalized estimation equation model by gaussian method

## Discussion

This study determined the retention of CPR knowledge and skills after hands-only bystander CPR training among non-healthcare professionals in Pakistan. The mean CPR knowledge score of the participants improved substantially after CPR training. In addition, participants were able to retain considerable knowledge and skills of CPR after 6 months of the initial training. We found out that gender, and prior CPR training were strongly associated with an increase in the CPR knowledge scores.

CPR knowledge and skill retention were tested after 6 months of the initial training. This duration reflects findings from previous literature which suggests that skill retention is usually till 6 months [18]. Evidence suggests that retention of theoretical CPR knowledge is higher than skills performance [19]. However, our study results showed that CPR skills retention was higher than knowledge. The greater retention of CPR skills in our study might be attributed to video-based CPR demonstration, scenario-based drills on manikins, feedback from the facilitator, and the flexibility to perform skills on multiple attempts during assessment.

The evidence affirms that CPR knowledge and skills fade over time but at different rates and various factors influence the retention of CPR knowledge and skills [20]. Previous studies have highlighted that ‘low dosage, high frequency’ refresher training, in which participants undergo brief but frequent CPR training sessions, improves the quality and retention of skills significantly [21, 22]. Healthcare systems that integrate CPR training into civic, work, or school activities offer a useful and effective means to assure retention [23].

A prospective, randomized intervention trial involving 874 prehospital cardiac arrest patients revealed that survival following prehospital cardiac arrest is more likely when witnessed, but not necessarily when bystander CPR is administered [24]. A multi-center study conducted in Pakistan showed that nearly 90% of OHCA patients are witnessed, yet only 2.3% received bystander CPR [25]. The high number of OHCA witness in Pakistan could be attributable to joint family system as most of the cases in this study occurred in residences, similar to what was seen in India [26]. However, nearly 50% of the witnessed cardiac arrest in European countries receive bystander CPR, particularly in public places [27, 28].

The ability to perform effective CPR is critical and a lifesaving skill for bystanders in line with the pre-hospital emergency care system. Previous studies have confirmed that learning bystander CPR can improve OHCA outcomes [29, 30]. Our study revealed that a combination of video and instruction-based CPR training is an effective method to improve the knowledge and skills of bystanders which is widely used in other contexts [31, 32] and had similar positive effects. In addition, implementing effective training methods to increase bystander CPR knowledge may improve resuscitation in high-risk groups [33, 34]. In a Swedish longitudinal study, efforts to provide conventional training significantly increased the number of individuals with CPR training and nearly doubled the proportion receiving bystander CPR from 31 to 55% in two decades [35]. In addition, previous research indicates that training targeted populations, such as neighbourhoods with poor bystander CPR rates, may be an effective strategy to increase bystander CPR rates and OHCA outcomes [36].

Bystander CPR is the first and foremost component in the chain of survival following cardiac arrest [37]. Based on the chain of survival that focuses on an integrated continuum of care; Pakistan lacks access to automated external defibrillators, a robust prehospital emergency care system, and health care facilities capable of both continuing resuscitative efforts and providing integrated and intensive post-resuscitative care and rehabilitation [38–40]. Even though, bystander CPR has gained widespread recognition as a life-saving skill; implementation of bystander CPR training and public access to it is generally limited in low-and-middle income countries (LMICs). However, in Pakistan, healthcare professionals have inadequate knowledge of CPR [41, 42]. There are several major concerns that bystanders may have regarding their readiness to perform CPR in Pakistan, including making a mistake when performing CPR or unintentionally injuring the patient, being concerned about legal consequences, and hesitating to perform CPR on the opposite gender which is consistent with the previous findings [43]. In addition, the reluctance to perform CPR on women is linked with fears of inappropriate touching and allegations of sexual assault [44]. This again underlines the importance of CPR training particularly with regards to bystander receptivity.

In this study, skill assessment was primarily done by master trainers. These are healthcare professionals who are BLS certified and have received prior CPR training. They are qualified to teach and evaluate laypersons who can act as providers in a community setting. Master trainers represent a sustainable model of community CPR training, leading to the ‘Train the Trainer’ models [45]. A pragmatic example of this within Pakistan is the Pakistan LifeSavers Program (PLSP) with the mission of training 10 million laypersons in 10 years [46].

### Limitations of the study

This study has various limitations. For one, it could not capture all participants for a refresher training session and so comparison of post-test and re-test results may depict a less than the accurate picture but is still consistent in terms of reasonable depreciation of knowledge after a considerable period. Secondly, there could be a difference in the rating consistency among facilitators because the co-faciltiators were less trained which may affect the inter-rater reliability. Also, there was a lack of participation from rural settings where access to care in case of emergencies is limited and where the need for such training would be greater. In addition, we were unable to evaluate the quality of the educational skills of trainers due to the time constraints of the instructors.

Future studies could focus on assessing baseline knowledge amongst participants from a variety of backgrounds and residential settings, both urban and rural areas. In addition, different methods of training delivery can be evaluated, particularly, video-based learning for refresher training and assessment of CPR skill retention.

## Conclusion

The bystander CPR training program did not only improve the knowledge and skills of the participants but also had a reasonable retention rate. This study may prove to be a steppingstone for researchers specially in the LMICs to explore interventions that may help to strengthen the chain of survival for patients with out of hospital cardiac arrest.

## Supplementary Information

**Additional file 1.** 

**Additional file 2.** 

## Data Availability

The datasets generated and analysed during the current study are not publicly available due to institutional policies on data sharing but are available from the corresponding author on reasonable request.

## Abbreviations

CPR: Cardiopulmonary Rescussitation
OHCA: Out of Hospital Cardiac Arrest
ROC: Resuscitation Outcomes Consortium Registry
CARES: Cardiac Arrest Registry to Enhance Survival
AED: Automated External Defibrillator
LMICs: Low-and-middle Income Countries
AKU: Aga Khan University
AHA: American Heart Association
GEE: Generalized Estimating Equation
PLSP: Pakistan LifeSavers Program
