# Supplementary material for: Out of hospital cardiac arrest: experience of a bystander CPR training program in Karachi, Pakistan
Source: BMC Emerg Med. 2022 Jun 3;22:93. doi: 10.1186/s12873-022-00652-2 (PMC9164717; doi:10.1186/s12873-022-00652-2)
Supplement: Supplementary file 1 — Additional file 1. [file 12873_2022_652_MOESM1_ESM.docx]

Additional File 1

**CPR knowledge assessment questionnaire**

**INSTRUCTION: PLEASE CIRCLE THE CORRECT ANSWERS**

**Demographic information:**

| 1. **Age (In years):** | | | | **۱۔ عمر(سال میں**) | | | |
| --- | --- | --- | --- | --- | --- | --- | --- |
| 1. **Gender:** | | | | **۲۔ جنس** | | | |
| a | Male | b | Female | لڑکی | b | لڑکا | a |
| 1. **Marital status:** | | | | ۳**۔ ازدواجی حیثیت** | | | |
| a | Single | b | Married | شادی شدہ | b | غیر شادی شدہ | a |
| c | Widow/Widower | d | Divorced | بیوہ /رنڈوا | d | طلاق یافتہ | c |
| 1. **Education** | | | | **تعلیم**  ۴۔ | | | |
| a | Primary school | b | Matric/ O-Level | میٹرک/ا و- لیول | b | پرائمری | a |
| c | Intermediate/ A-Level | d | Graduation | گریجویٹ | d | لیول A /انٹرمیڈیٹ | c |
| e | Post graduate | f | Others | یا کوئی اور | f | پوسٹ گریجویٹ | e |
|  | Specify others | | | دیگر کی وضا حت | | |  |
| 1. **Occupation** | | | | ۵**۔ پیشہ** | | | |
| a | Government Job | b | Private Job | پرائیویٹ جاب | b | گورنمنٹ جاب | a |
| c | Retired | d | Housewife | ہاؤس وائف | d | ریٹائرڈ | c |
| e | Self employed | f | Student | طالبعلم | f | کاروبار | e |
| g | Not working | h | Others | یا کوئی اور | h | کوئی کام نہیں کرتے | g |
|  | Specify others | | | دیگر کی وضا حت | | |  |

**Knowledge Assessment Questions:**

| 1. **What do you think “Cardiopulmonary resuscitation (CPR)” means?** | | **۱۔ آپ کے خیال میں سی پی آر (CPR)کا کیا مطلب ہے ؟** | |
| --- | --- | --- | --- |
| a | To apply strong compression to the chest at certain intervals | سینے کو ایک مخصوص وقفہ پر زور دینا | a |
| b | To compress the heart by directly opening the chest wall. | سینے کو کھول کر دل کا ڈائریکٹ مساج کرنا / دل کو دبانا | b |
| c | I have no idea | مجھے کوئی اندازہ نہیں ہے | c |
| 1. **Which of the following may be a sign of sudden heart arrest? (You can mark more than one option)** | | **۲۔ ان میں سے کونسی علامت اچانک دل بند ہونے کی علامت ہیں ؟**  **(ایک سے زیادہ منتخب کر سکتے ہیں)** | |
| a | Loss of consciousness | بےہوشی | a |
| b | No breathing/ Abnormal breathing | سانس کا نہ آنا یا رک رک کہ آنا | b |
| c | No Pulse | دل کی دھڑکن کا رک جانا /نبض کا نہ چلنا | c |
| d | A person sitting or standing suddenly collapsed | کسی شخص کا اچانک سے گر جانا | d |
| e | Chest pain | سینے / دل کا درد | e |
| f | I don’t know | میں نہیں جانتا/ معلوم نہیں | f |
| 1. **How can the consciousness state of the individual be determined? (You can choose more than one option)** | | ۳**۔ کسی شخص کے ہوش و حواس میں ہونے کا تعین کیسے لگایا جاسکتا ہے؟**  **(ایک سے زیادہ منتخب کر سکتے ہیں)** | |
| a | No response when called | جب آواز دیں تو جواب نا دیں | a |
| b | No response when tapped | تھپتھپانے / چھونے/ہلانے پہ حرکت نہ کرنا | b |
| c | Not moving at all | کوئی بھی حرکت نہ ہونا | c |
| d | None of the above | ان میں سے کوئی نہیں | d |
| 1. **How can the absence of respiration be determined? (You can choose more than one option)** | | **۴۔ سانس کے نا چلنے کا تعین کیسے لگایا جاسکتا ہے؟**  **(ایک سے زیادہ منتخب کر سکتے ہیں)** | |
| a | Not having any chest movement | سینے میں کوئی حرکت کا نہ ہونا | a |
| b | Not having any breath sounds | سانس چلنے کی آواز کا نہ آنا | b |
| c | Not able to feel their breath either from mouth or nose | محسوس کرنے پے منہ یا ناک سے سانس لینے کی آواز کا نہ آنا | c |
| d | I don’t know | میں نہیں جانتا/ معلوم نہیں | d |
| 1. **CPR is performed on which part of chest?** | | **۵۔ سینے کے کونسے حصّے پہ سی پی آر (CPR) کیا جاتا ہے ؟** | |
| a | Upper part of the chest | سینے کے اوپری حصّے پر | a |
| b | Middle of the chest | سینے کے درمیانے حصّے پہ | b |
| c | Lower of the chest | سینے کے نچلے حصّے پہ | c |
| d | I don’t know | میں نہیں جانتا/ معلوم نہیں | d |
| 1. **What must be the rate of the chest compressions?** | | **۶۔سی پی آر (CPR) میں سینے کو دبانے کی رفتار کتنی ہونی چاہیے ؟** | |
| a | At least 150 times per minute | کم سے کم 150 مرتبہ فی منٹ | a |
| b | At least 100 times per minute | کم سے کم 100 مرتبہ فی منٹ | b |
| c | At least 50 times per minute | کم سے کم 50 مرتبہ فی منٹ | c |
| d | I don’t know | میں نہیں جانتا/ معلوم نہیں | d |
| 1. **How much force must be applied during chest compressions?** | | **۷۔ سینہ دبانے کے** **لیے کتنی طاقت لگنی چاہیے ؟** | |
| a | The rib cage moves down ½ of the chest diameter | اتنی کہ پسلیاں ٢٢/ ١ گہرائی تک دب جائے | a |
| b | The rib cage moves down 1/3^rd^ of the chest diameter | اتنی کہ پسلیاں ۳/١١گہرائی تک دب جائے | b |
| c | The rib cage moves down ¼ of the chest diameter | اتنی کہ پسلیاں ۴/ ١ گہرائی تک دب جائے | c |
| d | As much force as possible | جتنی زیادہ طاقت لگائی جاسکتی ہے لگائی جائے | d |
| e | I don’t know | میں نہیں جانتا/ معلوم نہیں | e |
| 1. **The critical characteristics of high quality CPR include which of the following? (You can choose more than one option)** | | ۸۔ **اعلی میعار کے سی پی آر کی اہم خصوصیات میں نیچے دی گئی کونسی خصوصیات شامل ہیں ؟ (ایک سے زیادہ منتخب کر سکتے ہیں)** | |
| a | Starting chest compressions within 10 seconds of unresponsiveness. | مریض کے بے ہوش ہونے کے10 سیکنڈ کے اندر سینہ دبانا شروع کردیں | a |
| b | Pushing chest hard and fast | سینے کو زورسے اور تیزی سے دبانا | b |
| c | Minimizing interruptions while CPR | (CPR)سی پی آر کے دوران غیر ضروری وقفے سے گریز کریں | c |
| d | I don’t know | میں نہیں جانتا/ معلوم نہیں | d |
| 1. **How often should rescuers switch roles when performing two-rescuer CPR?** | | ۹۔ **مدد کرنے والے٢ 2لوگوں کو کتنی دیر میں ڈیوٹی تبدیل کرنی چاہے ؟** | |
| a | After every one minute | ہر ایک منٹ کے بعد | a |
| b | After every two minute | ہر دومنٹ کے بعد | b |
| c | After every three minute | ہر تین منٹ کے بعد | c |
| d | After every five minute | ہر پا نچ منٹ کے بعد | d |
| e | I don’t know | میں نہیں جانتا/ معلوم نہیں | e |
| 1. **Cardiopulmonary resuscitation (CPR) is an emergency procedure which is attempted to restart the heart that has stop beating?** | | 10۔ **سی پی آر (CPR)ایک ہنگامی طریقہ کار ہے جو دل کی حرکت کو بحال /دوبارہ شروع کر نے کے لیے کیا جاتا ہے ؟** | |
| a | Yes | ہاں | a |
| b | No | نہیں | b |
| c | I don’t know | میں نہیں جانتا/ معلوم نہیں | c |
| 1. **CPR has to be attempted always inside of a hospital ?** | | ۱۱۔  **سی پی آر(CPR) ہمیشہ ہسپتال کے اندر کرنا چاہیے ؟** | |
| a | Yes | ہاں | a |
| b | No | نہیں | b |
| c | I don’t know | میں نہیں جانتا/ معلوم نہیں | c |
| 1. **CPR is generally continued until the person regains consciousness or is declared dead or until AED/EMS arrives?** | | ۱۲۔ **عا م طور پر سی پی آر (CPR)اس وقت تک جاری رکھنا چاہیے جب تک مریض ہوش میں نہ آجاۓ یا موت کی تصدیق نہ ہوجاۓ یا اے ای ڈی (AED)(جھٹکا دینے والی مشین) / ہنگامی طبی امداد نہ پہنچ جائے؟** | |
| a | Yes | ہاں | a |
| b | No | نہیں | b |
| c | I don’t know | میں نہیں جانتا/ معلوم نہیں | c |
| 1. **The survival rate is high when CPR is done immediately؟** | | **۱۳۔ اگر سی پی آر (CPR) فوری شروع کردیا جائے تو زندگی بچنے کے امکانات/ چانس زیادہ ہوتے ہیں؟** | |
| a | Yes | ہاں | a |
| b | No | نہیں | b |
| c | I don’t know | میں نہیں جانتا/ معلوم نہیں | c |
| 1. **Is CPR effective in saving life of children?** | | ۱۴۔ کیا **سی پی آر (CPR) بچوں کی زندگی بچانے کے لیے فائدہ مند ہے ؟** | |
| a | Yes | ہاں | a |
| b | No | نہیں | b |
| c | I don’t know | میں نہیں جانتا/ معلوم نہیں | c |

**Attitude and practice Questions:**

| 1. **Have you ever seen CPR being performed?** | | **۱۔ کیا آپ نے کبھی سی پی آر (CPR) ہوتے ہوئے دیکھا ہے ؟** | |
| --- | --- | --- | --- |
| a | Yes | ہاں | a |
| b | No | نہیں | b |
| 1. **If yes then where?** | | **۲۔ اگر ہاں تو کہاں دیکھا ہے؟** | |
| a | Television | ٹیلی ویژن | a |
| b | Social media /Facebook | فیس بک یا سوشل میڈیا پہ | b |
| c | Real life scenario | حقیقی زندگی میں | c |
| d | Others | یا پھر کہیں اور | d |
| Please specify others | | کہیں اور کی وضاحت | |
| 1. **Do you know how to do CPR in the case of cardiac arrest (namely, sudden death)?** | | **۳ ۔ کیا آپکو پتا ہے** کہ **دل بند ہونے پر سی پی آر (CPR)کیسے کرتے ہیں؟** | |
| a | Yes | ہاں | a |
| b | No | نہیں | b |
| **اگر آپ کا جواب نہیں ہے تو سوال نمبر4,5,6,7 چھوڑدیں** | | | |
| 1. **Have you received any CPR training?** | | **۴۔ کیا آپنے کبھی سی پی آر (CPR)کی تربیت لی ہے؟** | |
| a | Yes | ہاں | a |
| b | No | نہیں | b |
| 1. **If your reply is yes to the above question, where did you receive the training?** | | **۵۔ اگر آپ کا جواب ہاں ہے تو کہاں سے لی ؟** | |
| a | At School | اسکول میں | a |
| b | At university | یونیورسٹی میں | b |
| c | During military service | ملٹری سروسز کے دوران | c |
| d | In Sports Club | سپورٹس کلب میں | d |
| e | At workplace | دفتر /کام کرنے کی جگه پہ | e |
| f | Television-Internet- Social media | ٹیلی ویژن یا انٹرنیٹ / سوشل میڈیا پہ | f |
| g | Others | یا پھر کہیں اور | g |
| Please specify others | | کہیں اور کی وضاحت | |
| 1. **What concerns may prevent you from doing Cardiopulmonary Resuscitation (CPR) if needed?** | | **۶۔ اگر آپ کو سی پی آر (CPR) کرنا پڑے تو کونسی وجوہات روک سکتی ہیں؟** | |
| a | Making a mistake while performing CPR / Lack of confidence | سی پی آر میں غلطی کا خدشہ /اعتماد کی کمی | a |
| b | It may harm the patient such as (break bones and damages internal organs | مریض کو نقصان پہنچنے کا خدشہ جیسا کہ ہڈی ٹوٹنا یا اندرونی اعضا کو نقصان پہنچنا | b |
| c | It may stop a working heart | کام کرتا / دھڑکتا ہوا دل بند ہوجانے کا خدشہ | c |
| d | Due to legal issues | قانونی مسائل کی وجہ سے | d |
| e | Contamination by blood/vomit or acquiring diseases | الٹی/خون یا کوئی بیماری لگ جانے کہ ڈر / خدشہ | e |
| f | Hesitation in performing CPR on an opposite gender (cultural issues) | دوسری جنس پہ سی پی آر کرنے میں جھجھک محسوس ہوگی | f |
| g | Security reasons | خود کی حفاظت کی وجہ سے | g |
| h | None | کوئی بھی نہیں | h |
| i | Hesitation in performing CPR on family member | خاندان کے کسی فرد پہ سی پی آر کرنے میں جھجھک/ ڈر محسوس کرنا | i |
| 1. **If cardiac arrest occurs in the following people, for whom would you conduct CPR? (You can choose more than one option)** | | **۷۔ اگر نیچے دیے گئےلوگوں میں کسی کا دل بند ہوجاۓ تو آپ کن پر سی پی آر (CPR) کرینگے؟ (ایک سے زیادہ منتخب کر سکتے ہیں)** | |
| a | Someone from the family | خاندان /فیملی کا کوئی بھی فرد | a |
| b | Your friend/someone you know/acquaintance | کوئی دوست/ کوئی جان پہچان والے | b |
| c | You neighbor | پڑوسی | c |
| d | Any stranger | کوئی اجنبی | d |
| e | Person who has poor personal hygiene lying on the road | سڑک پہ پڑا ہوا شخص جو دِِکھنے میں گندا/ میلا کچیلا ہو | e |
| f | All of the above | اوپر دیے گے تمام لوگ | f |
| g | A road traffic crash victim lying on the road | سڑک پہ پڑا ہوا شخص جوٹریفک حادثہ کا شکار ہو | g |
| 1. **If you have witnessed cardiac arrest, what will you do in such situation? (You can choose more than one option)** | | **۸۔ اگر آپکے سامنے کسی کا دل بند ہوجاۓ تو آپ کیا کریں گے؟**  **(ایک سے زیادہ منتخب کر سکتے ہیں)** | |
| a | I will start CPR | سی پی آر کرونگا /کرونگی | a |
| b | I will perform mouth to mouth breathing only and not do CPR | میں صرف منہ سے سانس دونگا / دونگی اور سی پی آر نہیں کرونگا/ کرونگی | b |
| c | I will check for response | میں دیکھوں گا/گی کہ وہ جواب دے رہا ہے کہ نہیں | c |
| d | I will call ambulance | ایمبولینس کو کال کرونگا /کرونگی | d |
| e | I will call for help | مدد کے لیے آواز لگاؤںگا / لگاؤں گی | e |
| f | I will check for scene safety | میں ا’س جگہ کی حفاظتی انتظامات دیکھونگا / دیکھونگی | f |
| 1. **When you encounter a person with cardiac arrest. What will be your first step after confirming scene safety?** | | **۹۔ اگر آپ کے سامنے کسی کا دل بندہوجاۓتو محفوظ جگہ کا تعین کرنے کے بعد آپ پہلی چیز کیا کرینگے ؟** | |
| a | Clear airway | سانس کی نا لی کھولوں گا/ کھولوں گی تاکہ ہو**ا/** سانس لینے کا راستہ بحال ہو سکے . | a |
| b | Check for response | چیک کرونگا / کرونگی کہ وہ جواب دے رہا ہے یا نہیں | b |
| c | Start CPR | سی پی آر شروع کرونگا/کرونگی | c |
| d | Call for help | مدد کے لیے آواز لگاؤں گا / لگاؤں گی | d |
| 1. **When you encounter a person in what appears to be cardiac arrest. What will be your second step?** | | **۱۰۔ اگر آپکے سامنے کسی کا دل بند ہوجانے تو دوسری چیز آپ کیا کرینگے؟** | |
| a | Clear airway | سانس کی نا لی کھولوں گا/ کھولوں گی تاکہ ہو**ا/** سانس لینے کا راستہ بحال ہو سکے . | a |
| b | Check for response | چیک کرونگا / کرونگی کہ وہ جواب دے رہا ہے یا نہیں | b |
| c | Start CPR | سی پی آر شروع کرونگا /کرونگی | c |
| d | Call for help | مدد کے لیے آواز لگاؤں گا / لگاؤں گی | d |
| 1. **When you encounter a person with cardiac arrest. What will be your third step?** | | **۱۱۔ اگر آپکے سامنے کسی کا دل بند ہوجانے تو تیسری چیز آپ کیا کرینگے؟** | |
| a | Clear airway | سانس کی نا لی کھولوں گا/ کھولوں گی تاکہ ہو**ا/** سانس لینے کا راستہ بحال ہو سکے . | a |
| b | Check for response | چیک کرونگا / کرونگی کہ وہ جواب دے رہا ہے یا نہیں | b |
| c | Check for breathing | دیکھونگا / دیکھونگی کہ سانس لے رہا ہے کے نہیں | c |
| d | Start CPR | سی پی آر شروع کرونگا /کرونگی | d |
| e | Call for help | مدد کے لیے آواز لگاؤں گا / لگاؤں گی | e |
| 1. **When you encounter a person with cardiac arrest. What will be your fourth step?** | | **۱۲۔ اگر آپکے سامنے کسی کا دل بند ہوجانے تو چوتھی چیز آپ کیا کرینگے؟** | |
| a | Clear airway | سانس کی نا لی کھولوں گا/ کھولوں گی تاکہ ہو**ا/** سانس لینے کا راستہ بحال ہو سکے . | a |
| b | Call for help | مدد کے لیے آواز لگاؤں گا / لگاؤں گی | b |
| c | Check for breathing | دیکھونگا / دیکھونگی کہ سانس لے رہا ہے کے نہیں | c |
| d | Start CPR | سی پی آر شروع کرونگا /کرونگی | d |
| e | Check for response | چیک کرونگا / کرونگی کہ وہ جواب دے رہا ہے یا نہیں | e |
