# Supplementary figures and images for: Out of hospital cardiac arrest: experience of a bystander CPR training program in Karachi, Pakistan
Source: BMC Emerg Med. 2022 Jun 3;22:93. doi: 10.1186/s12873-022-00652-2 (PMC9164717; doi:10.1186/s12873-022-00652-2)

Additional File 2

**Checklist for CPR Performance**


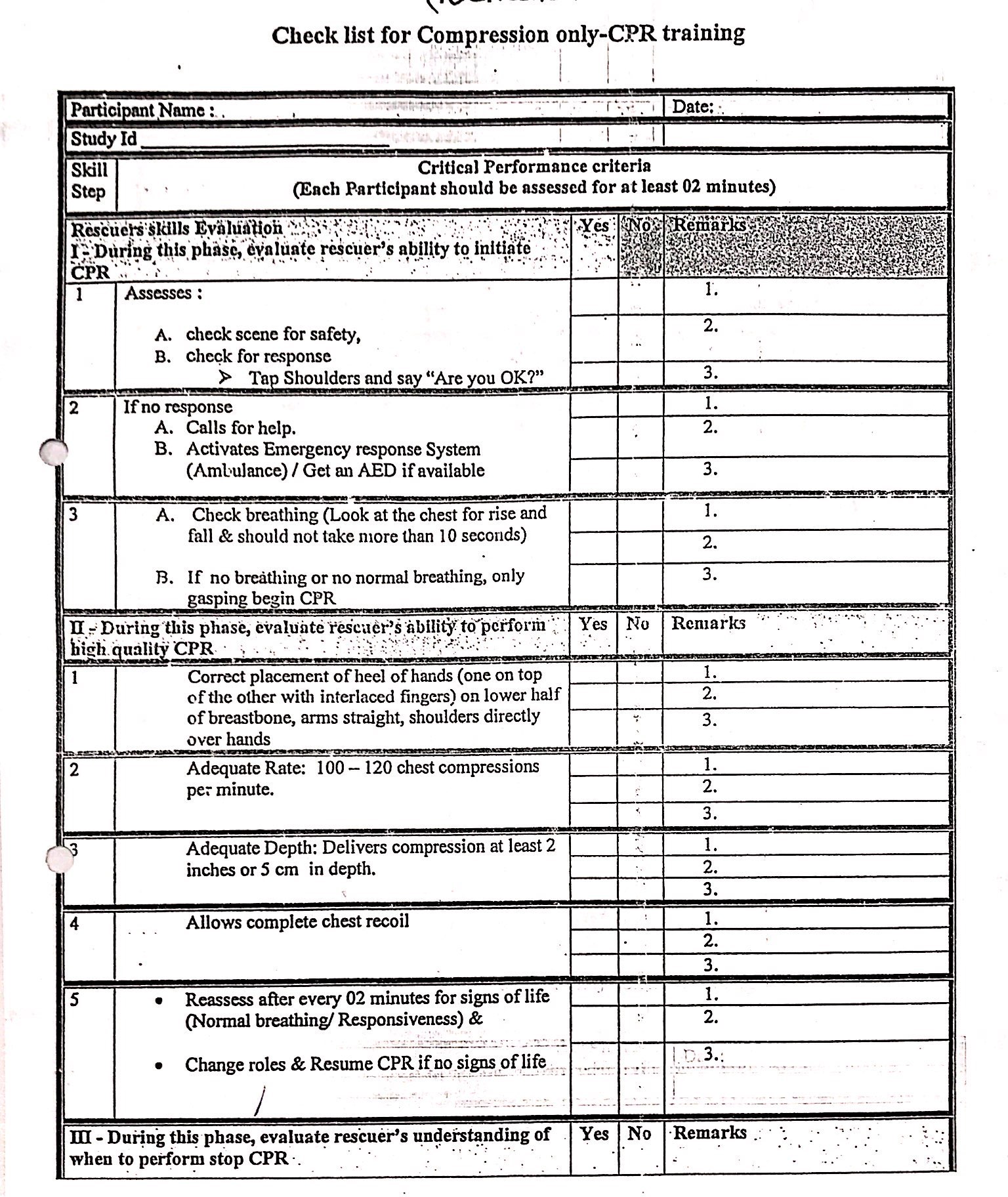

Supplement: Supplementary file 2 — Additional file 2. [file 12873_2022_652_MOESM2_ESM.docx]
